# Cerium Synchronous Doping in Anatase for Enhanced Photocatalytic Hydrogen Production from Ethanol-Water Mixtures

**DOI:** 10.3390/molecules28062433

**Published:** 2023-03-07

**Authors:** Mei-Hong Tong, Yan-Xin Chen, Tian-Ming Wang, Shi-Wei Lin, Gen Li, Qian-Qian Zhou, Rui Chen, Xia Jiang, Hong-Gang Liao, Can-Zhong Lu

**Affiliations:** 1 College of Chemistry, Fuzhou University, Fuzhou 350116, China; 2 CAS Key Laboratory of Design and Assembly of Functional Nanostructures, Fujian Provincial Key Laboratory of Nanomaterials, Fujian Institute of Research on the Structure of Matter, Chinese Academy of Sciences, Fuzhou 350002, China; 3 Xiamen Key Laboratory of Rare Earth Photoelectric Functional Materials, Xiamen Institute of Rare-Earth Materials, Haixi Institutes, Chinese Academy of Sciences, Xiamen 361021, China; 4 Fujian Science & Technology Innovation Laboratory for Optoelectronic Information of China, Fuzhou 350108, China; 5 State Key Laboratory of Physical Chemistry of Solid Surfaces, College of Chemistry and Chemical Engineering, Xiamen University, Xiamen 361005, China

**Keywords:** cerium-doped, TiO_2_ nanoparticles, lattice structure, photocatalytic, photoelectrochemical water splitting

## Abstract

Cerium element with a unique electric structure can be used to modify semiconductor photocatalysts to enhance their photocatalytic performances. In this work, Ce-doped TiO_2_ (Ce/TiO_2_) was successfully achieved using the sol-gel method. The structural characterization methods confirm that Ce was doped in the lattice of anatase TiO_2_, which led to a smaller grain size. The performance test results show that the Ce doped in anatase TiO_2_ significantly enhances the charge transport efficiency and broadens the light absorption range, resulting in higher photocatalytic performance. The Ce/TiO_2_ exhibited a photocurrent density of 10.9 μA/cm^2^ at 1.0 V vs. Ag/AgCl, 2.5 times higher than that of pure TiO_2_ (4.3 μA/cm^2^) under AM 1.5 G light. The hydrogen (H_2_) production rate of the Ce/TiO_2_ was approximately 0.33 μmol/h/g, which is more than twice as much as that of the pure anatase TiO_2_ (0.12 μmol/h/g). This work demonstrates the effect of Ce doping in the lattice of TiO_2_ for enhanced photocatalytic hydrogen production.

## 1. Introduction

H_2_ energy, with its high calorific value and environmental friendliness, has been regarded as an alternative to unsustainable fossil fuels with the most potential [1,2,3]. Solar to H_2_ conversion is a promising technique for solving the energy crisis and environmental problems caused by continuously consuming nonrenewable fossil fuels [4,5]. Since Fujishima and Honda first discovered the photoelectrochemical splitting of water into H_2_ and O_2_ using TiO_2_ photoanode [6,7,8,9], TiO_2_ has been widely applied in photocatalysis due to the fact of its low cost, stability, and nontoxicity [10,11,12]. However, TiO_2_ only reacts to ultraviolet light due to the fact of its wide band gap of 3.2 eV, resulting in low quantum efficiency. In addition, the high-rate recombination of photogenerated electron/hole pairs causes the practical application of TiO_2_ for photocatalytic H_2_ production to be strictly confined. To address these drawbacks and boost the solar energy utilization efficiency of TiO_2_, considerable efforts have been devoted. Many strategies, for instance, metal doping, such as Ta and Fe doping [13,14]; nonmetal doping, such as N and C doping [15,16]; surface dye sensitization [17]; and noble Ag and Au doping [10,18], is often employed to modulate the electronic structure and photocatalytic activity of TiO_2_. In particular, introducing rare earth elements into TiO_2_ can effectively improve the electron–hole separation and extend the visible light response of TiO_2_ [19,20]. Among rare earth elements, cerium (Ce) shows the variable valence states Ce^3+^/Ce^4+^ with different electronic structures (4f^1^5d^0^ and 4f^0^5d^0^, respectively), which result in the facile formation of oxygen vacancies. Thus, the shuttling of electrons between two valence states largely facilitates the charge separation and the following photocatalytic reactions [21,22]. However, most research on Ce-doped titanium dioxide focuses on photocatalytic degradation, with few studies on photocatalytic water splitting.

In this work, a simple sol-gel method achieved Ce synchronous doping in the lattice of TiO_2_. The morphology and structure of the pure TiO_2_, Ce-doped TiO_2_ (Ce/TiO_2_), and CeO_2_-mixed TiO_2_ (CeO_2_-TiO_2_) samples were observed by X-ray diffraction (XRD), scanning electron microscopy (SEM), and transmission electron microscopy (TEM). The PEC performances of TiO_2_, Ce/TiO_2_, and CeO_2_-TiO_2_ were comprehensively investigated. The electron capture center formed by Ce doping into the TiO_2_ lattice dramatically improves the separation efficiency of photogenerated electrons and holes. In addition, the narrowed band gap of the Ce-doped TiO_2_ shows excellent visible light absorption and photocurrent response. The Ce/TiO_2_ samples realized high photocurrent density and incident photon-to-current efficiency (IPCE) values thanks to the Ce doping. The Ce doping also hugely improved the photocatalytic H_2_ production of the TiO_2_ electrode. This work offers a practical strategy and significant reference for preparing and understanding efficient visible-light-activated rare-earth-doped photocatalysts.

## 2. Results

### 2.1. Structure and Morphology

**Figure 1:**
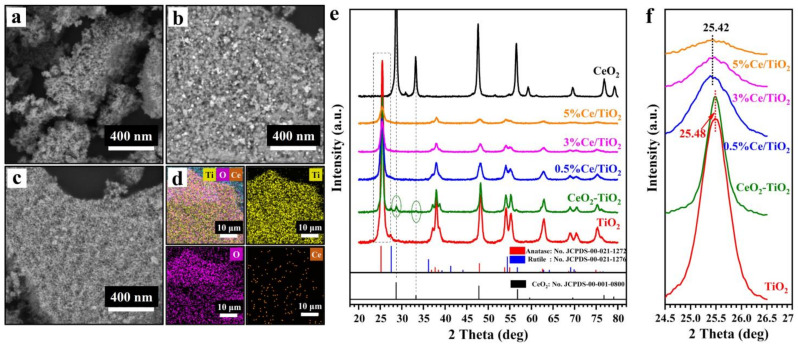
SEM images of (**a**) pure TiO_2_, (**b**) CeO_2_-TiO_2_, and (**c**) Ce/TiO_2_; (**d**) element mapping images of Ti, O, and Ce in the Ce/TiO_2_ sample; (**e**) XRD patterns of the CeO_2_, pure TiO_2_, CeO_2_-TiO_2_ mixture, and Ce/TiO_2_ samples with different Ce doping contents; (**f**) 23.5–25.0° refined XRD spectrums of (**e**).

The surface morphology of the pure TiO_2_, Ce-doped TiO_2_ (Ce/TiO_2_, 0.5% Ce), and CeO_2_-mixed TiO_2_ (CeO_2_-TiO_2_) samples were observed by SEM, and their crystal structures were identified by XRD measurement, as shown in Figure 1. The elemental mapping results of the as-prepared Ce/TiO_2_ sample demonstrated a uniform distribution of Ce in TiO_2_, as shown in Figure 1d. As shown in the SEM and EDS mapping images, Ce/TiO_2_ consists of TiO_2_ nanoparticles with a homogeneous distribution of Ce elements. The relative amount of the Ce element in Ce/TiO_2_ is close to that in CeO_2_-TiO_2_, as shown in Table S1.

The crystal structures of these obtained samples were identified by XRD, as shown in Figure 1e,f. The pure TiO_2_, CeO_2_-TiO_2_, and Ce/TiO_2_ powders comprised the anatase phase dominantly. The samples of pure TiO_2_ and CeO_2_-TiO_2_ still had a rutile phase with a peak at 27.4° corresponding to the (110) plane of the rutile TiO_2_. However, the growth of the rutile phase in the pure TiO_2_ was inhibited after doping with Ce. In addition, the peak intensity of the anatase TiO_2_ in the Ce-doped TiO_2_ samples decreased when the Ce content increased. Excessive Ce ions stay at the particle surface or the grain boundaries, suppressing the anatase crystal growth [23,24]. From the high-resolution plots of the prominent peak (101) at 25.48°, as shown in Figure 1b, the crystal sizes of the pure TiO_2_, 0.5%Ce/TiO_2_, 3%Ce/TiO_2_, and 5%Ce/TiO_2_ were calculated to be 13.92, 12.03, 12.09, and 12.14 nm, respectively, according to the Scherrer formula [22]. The peaks of the anatase (101) crystal plane in the Ce-doped TiO_2_ samples shifted towards a lower diffraction angle compared to those of the pure TiO_2_ and CeO_2_-TiO_2_. The interplanar spacing of the (101) crystal plane increased due to the Ce doping [24]. No prominent characteristic peaks of CeO_2_ were observed in the Ce-doped TiO_2_ samples due to the low doping contents.

**Figure 2:**
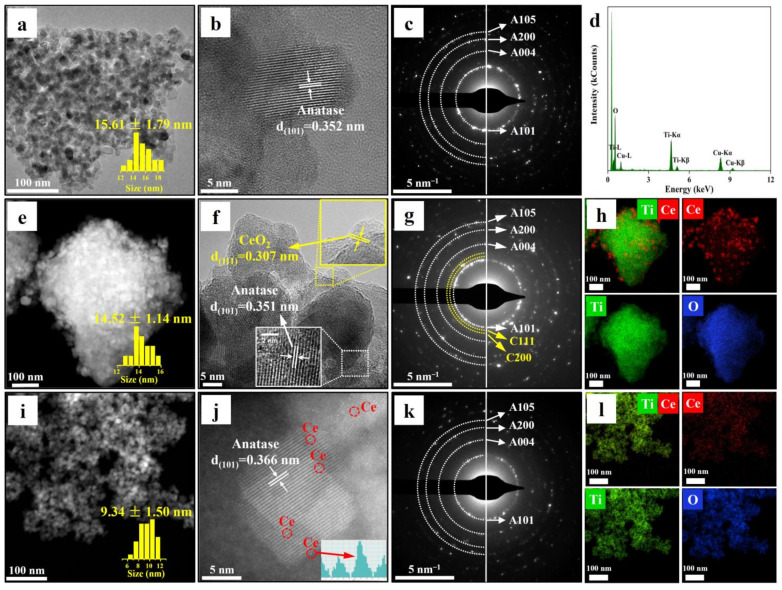
TEM images, HRTEM images, SAED patterns, and elemental mapping of (**a**–**d**) pure TiO_2_; (**e**–**h**) CeO_2_-TiO_2_; (**i**–**l**) Ce/TiO_2_, respectively.

As shown in Figure 2, the internal microstructures of the pure TiO_2_, Ce/TiO_2_ (0.5%Ce/TiO_2_), and CeO_2_-TiO_2_ were explored by TEM, HRTEM, SAED, and EDS mapping, respectively. As shown in the TEM images, the Ce/TiO_2_ consisted of TiO_2_ nanoparticles with a homogeneous distribution of Ce elements. The pure TiO_2_ mainly consisted of anatase nanoparticles, as shown in Figure 2a–d. The HRTEM image (Figure 2b) shows a lattice spacing of 0.350 nm, corresponding to the (101) plane of anatase. The composite of the CeO_2_-TiO_2_ nanoparticles, with an average size of 14.52 nm, aggregated together, as observed by TEM (Figure 2e,f). The HRTEM image (Figure 2f) of CeO_2_-TiO_2_ indicates the lattice lines of the anatase (101) plane and the crystal plane (111) of CeO_2_, highlighted by yellow boxes and enlarged in yellow boxes. Figure 2g shows both the anatase plane and the CeO_2_ plane. From the EDS mapping image, as shown in Figure 2h, it is clear that the Ce elements are distributed unevenly in the composite structure of CeO_2_-TiO_2_. However, in the sample of the Ce-doped TiO_2_ (0.5%Ce/TiO_2_), the nanoparticle size was smaller than that of the composite CeO_2_-TiO_2_, which is consistent with the results of the XRD and SEM. The HRTEM Image (Figure 2j) of Ce/TiO_2_ shows that the lattice spacing of the anatase (101) plane increased to 0.366 nm. In addition, several bright spots distributed in the anatase lattice are highlighted by the red circles, indicating Ce atoms doping into the lattice of the TiO_2_. The SAED patterns in Figure 2k also confirm the single crystallinity of the anatase TiO_2_ in the Ce-doped TiO_2_. The elemental mapping results shown in Figure 2l further verify the uniform distribution of Ce element in the lattice structure of TiO_2_ in Ce/TiO_2_. Therefore, the sol-gel method successfully doped the Ce in the lattice of TiO_2_.

### 2.2. Chemical Composition and Surface Information

**Figure 3:**
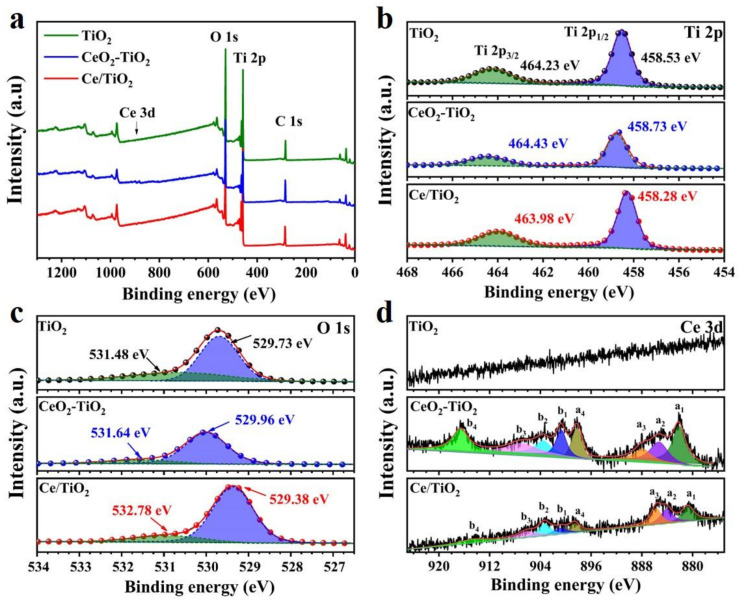
XPS spectra of the pure TiO_2_, Ce/TiO_2_, and CeO_2_-TiO_2_: (**a**) survey spectra; (**b**) Ti 2p; (**c**) O 1s; (**d**) Ce 3d.

For obtaining the chemical composition and valence state of the Ce-doped TiO_2_, X-ray photoelectron spectroscopy (XPS) measurements were conducted, as shown in Figure 3. The pure TiO_2_, Ce/TiO_2_, and CeO_2_-TiO_2_ were all composed of O, Ti, and C, as shown in the survey spectra (Figure 3a). The Ti 2p peaks at 458.28 eV and 463.98 eV are attributed to Ti 2p_3/2_ and Ti 2p_1/2_, respectively, indicating the Ti^4+^ state in the Ce/TiO_2_, as shown in Figure 3b. A slight shift in the Ti 2p in the Ce-doped TiO_2_ towards a lower value compared to that of pure TiO_2_ could be due to the interaction between Ti^4+^ and Ce^3+^ or Ce^4+^ species [25]. In Figure 3c, the O 1s peak at 529.73 eV corresponds to the lattice oxygen of TiO_2_, while the peak at 531.48 eV corresponds to the surface-adsorbed oxygen species [26]. The O 1s spectrum of the Ce/TiO_2_ had a smaller peak area at 532.78 eV compared to that of pure TiO_2_, which indicates a reduction in oxygen vacancies. In addition, the O 1s peak slightly shifted towards the lower binding energy region due to the Ce doping. The Ce 3d spectra (Figure 3d) show eight fitting peaks, where *a* and *b* correspond to the two spin orbitals (Ce 3d_5/2_ and Ce 3d_3/2_) in the Ce/TiO_2_, respectively [27]. The peaks *a*_2_ and *b*_2_ are attributed to Ce^3+^, while the others correspond to Ce^4+^. The relative content of Ce ions can be calculated from the ratio of the fitting peak area. The relative content of Ce^3+^ in the Ce/TiO_2_ (30.4%) was higher than that of the CeO_2_-TiO_2_ (24.6%), indicating that more Ce^3+^ ions were retained in the synchronous doping process. The higher content of Ce^3+^ in Ce/TiO_2_ plays a key role in the formation of oxygen defects and improves the redox conversion between Ce^3+^ and Ce^4+^ [28].

**Figure 4:**
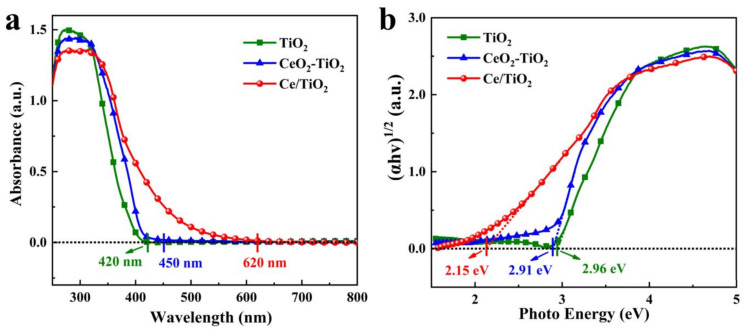
(**a**) UV-Vis absorbance spectra and (**b**) the corresponding Tauc plot of the pure TiO_2_, CeO_2_-TiO_2_, and Ce/TiO_2_.

The UV-Vis absorbance spectra in Figure 4a show the redshift of the absorption edge to the visible region in TiO_2_ after Ce doping, which may be due to the Ce 4f intermediate energy level inside the band gap of TiO_2_ [25]. The Tauc plot extracted from the UV-Vis absorbance spectra exhibits the band gap of the samples, as shown in Figure 4b. The band gaps of the pure TiO_2_, CeO_2_-TiO_2_, and Ce/TiO_2_ were 2.96 eV, 2.91 eV, and 2.15 eV, respectively.

For further study of the separation of the photogenerated electron/hole pairs, fluorescence (FL) spectroscopy was conducted, as shown in Figure S1. The luminous signal was generated in the region of 375–450 nm, with the longest signal at 420 nm, indicating the generation of * OH radicals in the reaction process. Generally, a higher intensity of FL suggests a higher recombination of the photogenerated carriers [29]. Pure TiO_2_ had a stronger spectral signal than that of the Ce-doped TiO_2_, indicating the high recombination rate of the photogenerated carriers in the pure TiO_2_. The Ce doping effectively improved the charge separation in the TiO_2_. However, the FL intensities of 3% Ce/TiO_2_ and 5% Ce/TiO_2_ increased compared to that of the 0.5% Ce/TiO_2_. The excessive Ce doping could form a new electron–hole recombination center, resulting in a lowered efficiency of charge separation. Therefore, 0.5% of Ce doping in TiO_2_ could provide better efficiency for charge separation and photocatalytic reactions compared to pure TiO_2_ and other Ce-doped TiO_2_ samples with high Ce doping contents.

## 3. Discussion

### 3.1. Photoelectrochemical Performance

**Figure 5:**
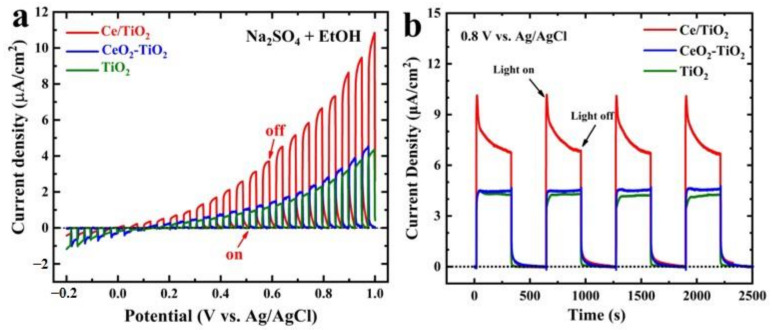
(**a**) Chopped linear sweep voltammetry (LSV) curves of the pure TiO_2_, CeO_2_-TiO_2_, and Ce/TiO_2_; (**b**) chronoamperometry data plots of the pure TiO_2_, CeO_2_-TiO_2_, and Ce/TiO_2_ at 0.8 V vs. Ag/AgCl.

The PEC performance of these as-prepared samples was further investigated. Under chopping irradiation, a linear sweep voltammetry (LSV) measurement was carried out under AM 1.5 G light, as presented in Figure 5a. Ce/TiO_2_ shows a photocurrent density of 10.9 μA/cm^2^ at 1.0 V vs. Ag/AgCl, which is 2.5 and 2.4 times higher than that of pure TiO_2_ (4.3 μA/cm^2^) and CeO_2_-TiO_2_ (4.5 μA/cm^2^), respectively. The highly increased photocurrent of Ce/TiO_2_ mainly originates from the efficient charge separation. Ce doping in TiO_2_ enhances the efficiency of photogeneration and separation of electron-hole pairs, resulting in a high photocurrent response of Ce/TiO_2_. Figure S2 presents the chopped LSV curves of the as-prepared TiO_2_ samples, which were modified with different Ce loadings or were annealed at different temperatures.

Chronoamperometry measurement was used to investigate the chemical stability of the samples at 0.8 V vs. Ag/AgCl, as presented in Figure 5b. The CeO_2_-TiO_2_ showed a higher photocurrent density than the pure TiO_2_, which resulted from the heterojunction between TiO_2_ and CeO_2_. The maximum photocurrent densities of the pure TiO_2_, CeO_2_-TiO_2_, and Ce/TiO_2_ were 3.2, 4.5, and 8.0 μA/cm^2^ at 0.8 V vs. Ag/AgCl, respectively. These results indicate that Ce doping into the crystal lattice of TiO_2_ is beneficial to the separation of photogenerated electrons and holes. Ce ions in TiO_2_ form an intermediate energy level, which results in the formation of oxygen vacancies. Under light radiation, the absorbed oxygen on the surface of the photocatalyst generates hydroxyl radicals and superoxide radicals, improving the photocatalyst’s activity. The Ce/TiO_2_ sample exhibited steady photocurrent densities during the long cycling, implying that its photostability significantly improved.

**Figure 6:**
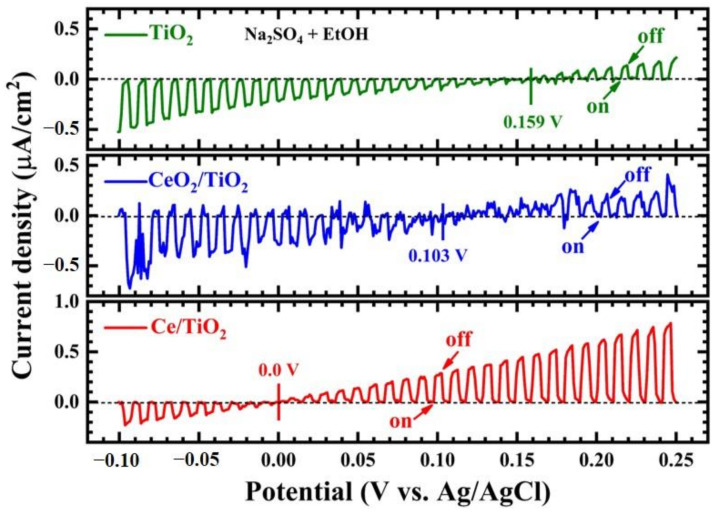
LSV curves for the onset potential from the low-potential region of the pure TiO_2_, CeO_2_-TiO_2_, and Ce/TiO_2_.

The onset potential of the photocurrents indicates the photocatalytic activity and the flat band potential of the photoanodes. A high onset potential implies a low utilization efficiency of solar energy [30]. As shown in Figure 6, the onset potential of the Ce/TiO_2_ (0.0 V) was more negative compared to that of the pure TiO_2_ (0.159 V) and CeO_2_-TiO_2_ (0.103 V). The low onset potential of the Ce/TiO_2_ indicates an efficient carrier transfer process in the Ce-doped TiO_2_ [31].

**Figure 7:**
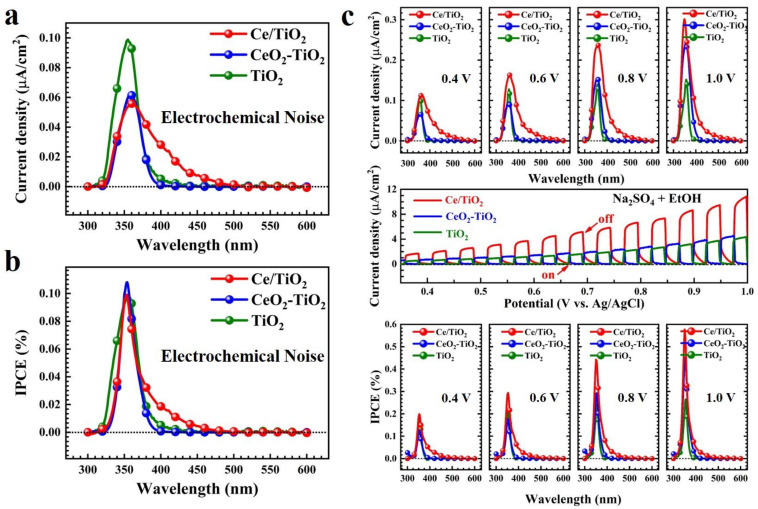
(**a**) Photocurrent density versus the monochromatic light in the electrochemical noise mode; (**b**) derived IPCE (%) spectra from the photocurrent density versus the monochromatic light; (**c**) photocurrent density and IPCE (%) spectra versus the monochromatic light at an increased applied voltage (V vs. Ag/AgCl).

The photocurrent densities versus the monochromatic light of the samples were measured in electrochemical noise (ECN) mode, which is a nondestructive and in situ monitoring technique to investigate the spontaneous electrochemical reactions of photoanodes [16,32], as shown in Figure 7a. The photocurrent density of the Ce/TiO_2_ was slightly lower than that of the pure TiO_2_ under the UV light region. It is worth noting that the Ce/TiO_2_ showed visible light absorption up to 500 nm, while the pure TiO_2_ and CeO_2_-TiO_2_ exhibited no obvious response under the visible light region. The successful Ce doping highly improves the visible light utilization efficiency of TiO_2_. The incident photon-to-current efficiencies (IPCEs) spectra were obtained from the photocurrent–wavelength curves, as shown in Figure 7b. The Ce/TiO_2_ had the highest IPCE value of 0.10% in the region of 350 nm and an obvious visible light response. The Ce/TiO_2_ still exhibited an IPCE value of 0.02% at 400 nm. The photocurrents and IPCE versus monochromatic light of Ce/TiO_2_ under applied voltages are shown in Figure 7c. The photocurrent response of the Ce/TiO_2_ increased with bias, which is consistent with the results of the LSV curves.

**Figure 8:**
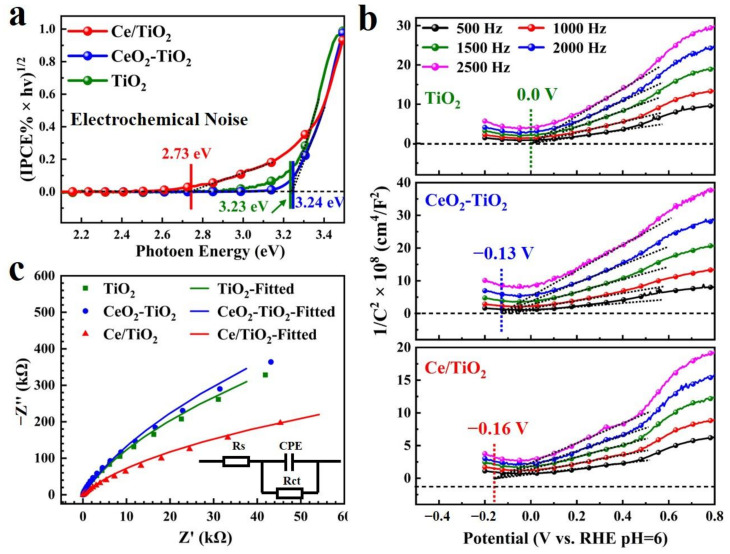
(**a**) The band gap determination extracted from the IPCE spectra as a function of (IPCE% × hv)^1/2^ vs. hv; (**b**) Mott–Schottky plots; (**c**) EIS Nyquist plots with the fitted equivalent circuit.

The band gap energy *Eg* of these samples can be calculated from the IPCE spectra by a Tauc plot of (IPCE % × hv)^1/2^ versus photon energy (hv) [16,33,34,35], as illustrated in Figure 8a. The band gaps of the pure TiO_2_, CeO_2_-TiO_2_, and Ce/TiO_2_ were 3.23, 3.24, and 2.73 eV, respectively. The band gap of the pure TiO_2_ is consistent with the reported data. The Ce/TiO_2_ had a lower Eg than pure TiO_2_ owing to the formation of oxygen defect levels above the valence band of TiO_2_. Thus, Ce doping in TiO_2_ narrows the band gap and enhances the visible light absorption of TiO_2_.

The charge transport in the pure TiO_2_, CeO_2_-TiO_2_, and Ce/TiO_2_ was also studied by electrochemical impedance spectroscopy (EIS), as shown in Figure 8c. The Ce/TiO_2_ sample shows a smaller arc in the Nyquist plot, indicating a lower charge transfer resistance at the electrode interface [36]. Ce doping introduces a new electronic state within the band gap of TiO_2_ near the conduction band, facilitating the charge separation and reducing the recombination rate of photogenerated electron/hole pairs.

Furthermore, the Mott-Schottky (M-S) measurement was used to determine the flat band potential (V_fb_) of these samples, as illustrated in Figure 8b. The curve slopes of the three samples are all positive, indicating their n-type semiconductor properties. The flat band potential can be regarded as the conduction band (CB) potential of n-type semiconductors, which is calculated by intercepting the slopes with the potential axis according to the M-S equation [25,34]. The values of V_fb_ of pure TiO_2_, CeO_2_-TiO_2_, and Ce/TiO_2_ are about 0.0, −0.13, and −0.16 V vs. RHE, respectively. The negative shift in the flat band potential of the Ce/TiO_2_ compared to the pure TiO_2_ implies a decrease in the transfer energy barrier of the interfacial electrons and the charge transfer resistance after Ce doping in TiO_2_ [35]. In addition, the slope of the M-S plot of the Ce/TiO_2_ is significantly smaller than that of the pure TiO_2_, indicating an increase in the charge carrier density according to the M-S equation.

### 3.2. PC H_2_ Evolution Performance and Mechanism for Water Splitting under Solar Light

**Figure 9:**
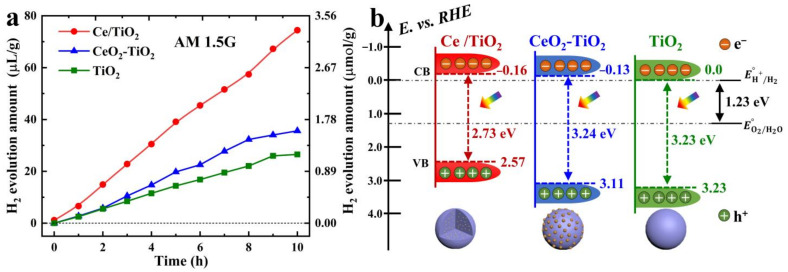
(**a**) Photocatalytic hydrogen evolution of the pure TiO_2_, CeO_2_-TiO_2_, and Ce/TiO_2_; (**b**) proposed band structures of the pure TiO_2_, CeO_2_-TiO_2_, and Ce/TiO_2_.

The photocatalytic H_2_ evolution of the pure TiO_2_, CeO_2_-TiO_2_, and Ce/TiO_2_ was evaluated under simulated solar light, as shown in Figure 9a. The H_2_ evolution rate of Ce/TiO_2_ was approximately 0.33 μmol/h/g, which is more than twice that of the pure TiO_2_ (0.12 μmol/h/g) and CeO_2_-TiO_2_ (0.16 μmol/h/g). Moreover, Ce/TiO_2_ also showed superior photostability for the photocatalytic H_2_ evolution over 10 h. The good performance in the water splitting of the Ce/TiO_2_ resulted from the narrow band gap and the effective separation of the photogenerated carriers. Ce doping in the lattice of TiO_2_ highly improves the photocatalytic performance of TiO_2_. The unique energy level structure of the Ce element provides doping levels in TiO_2_, facilitating electron transfer and charge separation.

Based on the above results, the mechanism of Ce doping in TiO_2_ for an improved photocatalytic performance was proposed. The energy band diagram of the pure TiO_2_, CeO_2_-TiO_2_, and Ce/TiO_2_ is illustrated in Figure 9b. Ce doping in the lattice of TiO_2_ narrows the band gap of TiO_2_, resulting in a visible-light response. The negatively shifted conduction band in Ce/TiO_2_ improves the carrier transfer process and reduces the recombination of photogenerated electron/hole pairs. Therefore, Ce/TiO_2_ exhibits a high photocatalytic performance for H_2_ evolution from water splitting.

## 4. Materials and Methods

### 4.1. Materials and Synthesis Methods

In the experiment, tetrabutyl titanate (TBOT), ethanol absolute, acetic acid, cerium nitrate hexahydrate (Ce(NO_3_)_3_·6H_2_O), and cerium oxide (CeO_2_) were all of analytical grade and purchased from commercial sources. Deionized water was used in all experiments.

The process of sample preparation was conducted using the sol-gel method. Firstly, the mixed solution of tetrabutyl titanate (8.5 mL) and ethanol absolute (20 mL) was prepared under magnetic stirring and recorded as solution A. Solution B, which contained 8.5 mL of deionized water, 7.5 mL of acetic acid, 20 mL of ethanol absolute, and a specific amount of Ce(NO_3_)_3_·6H_2_O, was prepared. Then, solution B was slowly dropped into the vigorously stirred solution A at a rate of one drop every 2 s, resulting in the formation of TiO_2_ sol, which was further heated at 70 °C for 4 h and dried at 70 °C for 10 h. Finally, the above gel was annealed at 500 °C for 5 h in the air. The obtained powders were marked as X% Ce/TiO_2_, where “X” represents the theoretical molar percentage of Ce in the product (0.5, 3.0, and 5.0%).

**Scheme 1:**
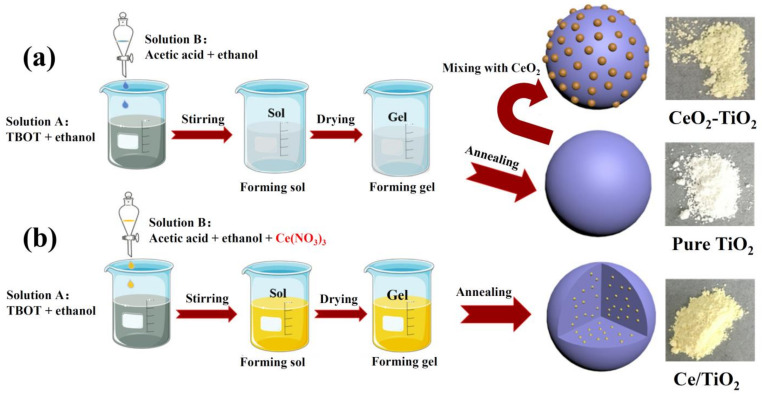
Synthesis diagram of (**a**) pure TiO_2_ and CeO_2_-TiO_2_; (**b**) Ce/TiO_2_.

For convenience, the Ce/TiO_2_ label mentioned below stands for the sample of 0.5% Ce/TiO_2_ with the best performance by default. TiO_2_ without Ce was prepared under the same conditions. For comparison, the TiO_2_ powders obtained by the sol-gel method were mixed with commercial CeO_2_ powders in a certain proportion (0.5% Ce), and the resulting powders were designated as CeO_2_-TiO_2_. The sample synthesis diagram is illustrated in Scheme 1.

### 4.2. Electrochemistry (EC) and Photoelectrochemical (PEC) Testing

All EC and PEC performances of the Ce/TiO_2_, CeO_2_-TiO_2_, and pure TiO_2_ were carried out in a standard three-electrode configuration with a side quartz window in which a mixture solution of 0.1 M Na_2_SO_4_ and ethanol (1:1 in volume, pH = 6.5) was the supporting electrolyte, and it was deaerated by bubbling high-purity Ar for 15 min before the EC/PEC measurements. A platinum plate and an Ag/AgCl electrode (saturated KCl) served as the counter electrode and reference electrode, respectively.

The working electrodes were fabricated on fluorine-doped conductive (FTO) glass plates (30 × 35 × 2.2 mm) for the PEC measurements. After being sonicated in soap suds and acetone, the FTO glass plates were rinsed with deionized water and then dried in an N_2_ atmosphere. A mixed solution, including 5 mg of the as-prepared photocatalyst powders, 1 mL of absolute ethanol, and 20 μL of 5 wt.% naphthol solution, was sonicated for 90 min and used for spinning casting onto the conductive surface of the FTO glass plates with a speed of 3000 r/min. The exposed area of the FTO glass was controlled to 1.0 cm^2^ (10 × 10 mm) by utilizing tape. After, the electrodes were dried at 80 °C for 2 h.

The linear sweep voltammetry (LSV) and chronoamperometry (I-t) curves of the samples were carried out in a three-electrode side window electrolytic cell, which was measured on a CHI760E electrochemical workstation under a high uniformity integrated Xenon light source (PLS-FX300HU, Beijing Perfectlight, Beijing, China) with an AM 1.5 G filter (100 mW/cm^2^).

An LSV measurement was typically carried out over a potential range of −0.2 to 1.0 V (vs. Ag/AgCl), and the scanning speed was 5 mV s^−1^ with the light cut off by a 5 s^−1^ shutter. An I-t measurement was performed under alternating illumination at 0.8 V (vs. Ag/AgCl). During the test, the working electrode was guaranteed to have an illuminated area of 1 cm^2^ and completely submerged in the electrolyte. The calculated voltage can be changed to a reversible hydrogen electrode (RHE) scale using the Nernst equation:

(1) $$E_{\mathrm{RHE}}=E_{\mathrm{Ag/AgCl}}+0.0592\times \mathrm{pH}+E_{\mathrm{Ag/AgCl}}^{0}$$

where $E_{\mathrm{Ag/AgCl}}^{0}$ = 0.1976 V vs. Ag/AgCl at room temperature.

The incident photo-to-current efficiency (IPCE) test system was composed of an electrochemical workstation (CS350H, CorrTest, Wuhan, China), a 300 W xenon lamp light source (PLS-SXE300D, Beijing Perfectlight, Beijing, China), and a grating monochromator (7ISU, SOFN Instruments Co., Ltd., Beijing, China) with filters to remove higher order diffraction, which is calculated using the following equation:

(2) $$\mathrm{IPCE}\left(\%\right)=\frac{1240\times J}{\lambda \times I_{0}}\times 100\%$$

where *J* is the photocurrent density measured at a specific wavelength, *λ* is the specific wavelength, and *I*_0_ is the intensity of the incident light.

Electrochemical impedance spectroscopy (EIS) and Mott-Schottky (M-S) plots were measured in the dark, using an electrochemical workstation (Squidstat Plus, Admiral Instruments, Tempe, AZ, USA). The fitted circuit can be obtained from the EIS curve, where the CPE element is calculated. The impedance of the CPE in an AC circuit is:

(3) $$\mathrm{CPE}=\sigma \omega^{-m}\left[\cos\left(\frac{m\pi }{2}\right)-j\sin\left(\frac{m\pi }{2}\right)\right]$$

where *σ* is the prefactor of the CPE, *ω* is the angular frequency, *m* is the CPE index (0 ≤ *m* ≤ 1), and *j* is an imaginary number $\left(j=\sqrt{-1}\right)$; if *m* = 1, then the CPE denotes the ideal capacitor C. The electrochemical analyzer works over a frequency range of 0.01 to 100,000 Hz, with voltage increments of 0.005 V and an AC amplitude of 10 mV. In a typical M-S measurement, the working electrodes were tested at 500, 1000, 1500, 2000, and 2500 Hz.

### 4.3. Photocatalytic (PC) Performances Testing

The PC splitting of the ethanol-water mixtures to generate the H_2_ measurement was carried out in the MCP-WS1000 photochemical workstation (Beijing Perfectlight Technology Co., Ltd., Beijing, China) equipped with a 50 mL quartz-made reactor under artificial solar irradiation. Specifically, 20 mg of as-prepared photocatalysts was ultrasonically dispersed (30 min) into a solution consisting only of 15 mL ethanol and 15 mL water (pH = 7), which were deaerated by vacuuming for 10 min before the H_2_ evolution measurements. The full spectrum source was simulated sunlight consisting of 9 LED lamps (365, 385, 420, 450, 485, 535, 595, and 630 nm and one white light LED, which was 420–750 nm), and the power of the total light irradiation was approximately 100 mW/cm^2^. The visible light source consisted of 9 white light LED lamps (420–750 nm) with a total visible light irradiation power of 100 mW/cm^2^. The temperature of the reaction was controlled by circulating condensed water at 5 °C throughout the reaction process using a water-cooling system. The produced hydrogen was calculated by the external standard method using the peak area obtained by the PLD-CGA1000 composite gas analyzer (Beijing Perfectlight Technology Co., Ltd., Beijing, China).

### 4.4. Materials Characterization

The morphology of the samples was observed by a scanning electron microscope with an operating voltage of 10 kV (SEM, Apreo S LoV ac, Thermo Fisher Scientific, Waltham, MA, USA).

The X-ray diffraction data were taken using an X-ray diffractometer with CuK radiation and a measurement range of 20–80°, (XRD, Miniflex 600, Akishima, Rigaku, Tokyo, Japan).

The transmission electron microscopy and selected area electron diffraction images were obtained from an FEI Talos F200s transmission electron microscope, operated at 200 kV (TEM, SAED, Thermo Fisher Scientific, Waltham, MA, USA).

High-resolution transmission electron microscopy was carried out on an FEI Titan Themis Z G3 30–300 spherical aberration-corrected transmission electron microscope, operated at 300 kV, equipped with both image and probe aberration (HRTEM, Thermo Fisher Scientific, Waltham, MA, USA).

X-ray photoelectron spectroscopy was used to characterize the atomic composition and state at the surface of the samples, using Al Ka rays as the excitation source (XPS, Thermo Scientific K-Alpha, Waltham, MA, USA).

The UV-Vis absorbance spectra were collected by a Cary 5000 spectrophotometer, (UV-Vis, Agilent, Santa Clara, CA, USA).

The photoluminescence spectra were acquired using a fluorescence spectroscopy test system (PL, FLS980, Edinburgh Instruments Ltd., Livingston, UK).

## 5. Conclusions

Ce synchronous doping in the lattice of anatase TiO_2_ was successfully achieved using a facile sol-gel method. The Ce doping suppressed the rutile phase and crystal growth of the anatase TiO_2_, leading to smaller grain size and a higher specific surface area compared to pristine TiO_2_. Ce doping in the lattice structure of TiO_2_ was also demonstrated by HRTEM and XPS. The charge recombination in the TiO_2_ was successfully reduced by Ce doping. The Ce-doped TiO_2_ showed a negative shift in the flat band potential and a lower carrier transport resistance. In addition, Ce doping narrowed the band gap of TiO_2_, leading to a visible light response. Therefore, Ce-doped TiO_2_ exhibits an enhanced photocurrent density and an excellent photocatalytic performance. The Ce-doped TiO_2_ showed superior photocatalytic performance for H_2_ production under solar light. Rare earth elements have great potential in the application of solar energy conversion. Ce doping in the lattice of TiO_2_ provides a new strategy for designing photocatalysts for photocatalytic water splitting.

## Data Availability

Not applicable.

## Supplementary Materials

The following supporting information can be downloaded at: https://www.mdpi.com/article/10.3390/molecules28062433/s1. Figure S1. Fluorescence spectra of the pure TiO_2_, 0.5%Ce/TiO_2_, 3%Ce/TiO_2_, and 5%Ce/TiO_2_; Figure S2. Chopped Linear sweep voltammetry (LSV) curves in 0.1M Na_2_SO_4_ under AM 1.5 G light: (a) pure TiO_2_, Ce-doped TiO_2_, and CeO_2_-TiO_2_; (b) 3%Ce/TiO_2_ annealed under different temperatures; Table S1. The relative amount of Ce element obtained from the ICP test.

## Sample Availability

Samples of the compounds are available from the authors.
