# Supplementary material for: Cerium Synchronous Doping in Anatase for Enhanced Photocatalytic Hydrogen Production from Ethanol-Water Mixtures
Source: Molecules. 2023 Mar 7;28(6):2433. doi: 10.3390/molecules28062433 (PMC10057969; doi:10.3390/molecules28062433)
Supplement: Supplementary file 1 [file molecules-28-02433-s001.zip › molecules-2251446-supplementary.pdf]

Supporting Information

Cerium Synchronous Doping in Anatase for Enhanced Photocatalytic Hydrogen Production from Ethanol–Water Mixtures

Mei-Hong Tong [1,2,4], Yan-Xin Chen [1,2,4,5,*], Tian-Ming Wang [2,4], Shi-Wei Lin [2,4], Gen Li [3], Qian-Qian Zhou [2,4], Rui Chen [2,4], Xia Jiang [2,4], Hong-Gang Liao [3*] and Can-Zhong Lu [2,4,5,*]

[1] College of Chemistry, Fuzhou University, Fuzhou, 350116, China
[2] CAS Key Laboratory of Design and Assembly of Functional Nanostructures, and Fujian Provincial Key Laboratory of Nanomaterials, Fujian Institute of Research on the Structure of Matter, Chinese Academy of Sciences, Fuzhou 350002, China
[3] State Key Laboratory of Physical Chemistry of Solid Surfaces, College of Chemistry and Chemical Engineering, Xiamen University, Xiamen 361005, China
[4] Xiamen Key Laboratory of Rare Earth Photoelectric Functional Materials, Xiamen Institute of Rare-Earth Materials, Haixi Institutes, Chinese Academy of Sciences, Xiamen 361021, China
[5] Fujian Science & Technology Innovation Laboratory for Optoelectronic Information of China, Fuzhou 350108, China
* Correspondence: yanxinchen@fjirsm.ac.cn (Y.-X.C.); hgliao@xmu.edu.cn (H.-G.L.); czlu@fjirsm.ac.cn (C.-Z.L.)

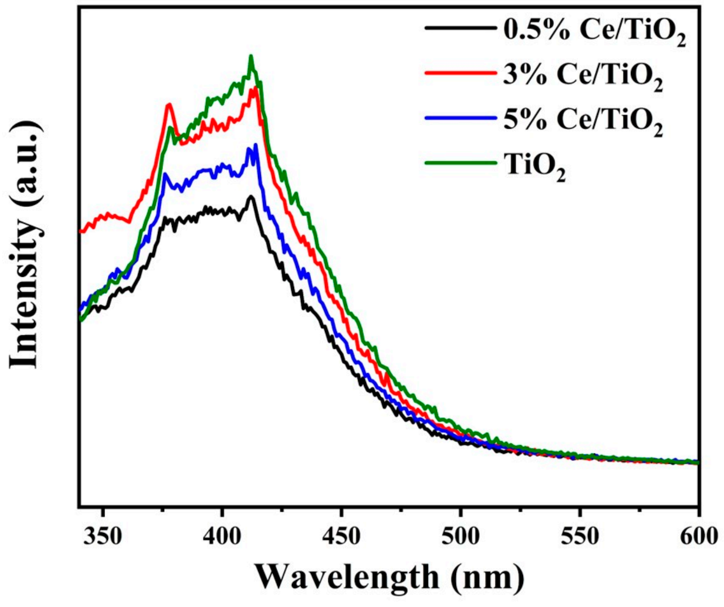

Figure S1. Fluorescence spectra of pure TiO_2, 0.5%Ce/TiO_2, 3%Ce/TiO_2, and 5%Ce/TiO_2.

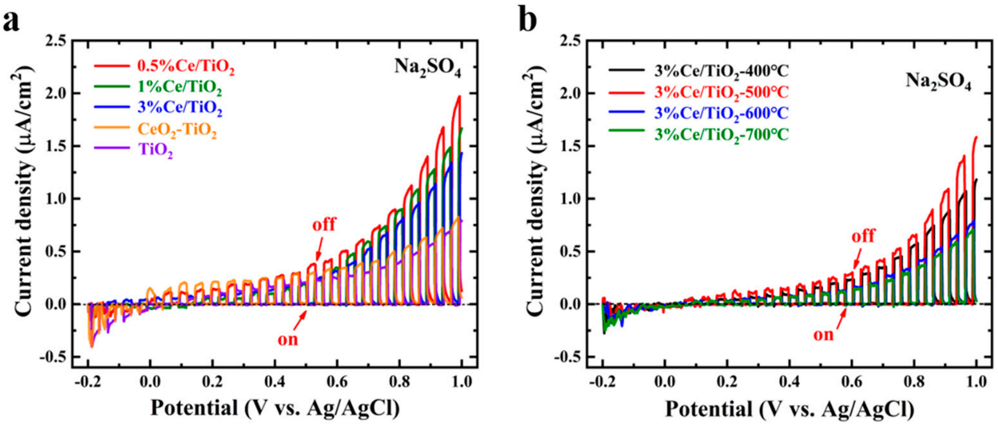

Figure S2. Chopped Linear sweep voltammetry (LSV) curves in 0.1M Na_2SO_4 under AM 1.5G light. (a) pure TiO_2, Ce-doped TiO_2 and CeO_2-TiO_2, (b) 3%Ce/TiO_2 anneared under different temperatures.

Table S1. The relative amount of Ce element obtained from ICP test.

Sample	Ti (ppb)	Ce (ppb)	atomic ratio of Ce (%)
Ce/TiO_2	1523	21	0.472
CeO_2-TiO_2	1917	29	0.517
